# Protective Effects of Pituitary Adenylate Cyclase-Activating Polypeptide and Vasoactive Intestinal Peptide Against Cognitive Decline in Neurodegenerative Diseases

**DOI:** 10.3389/fncel.2020.00221

**Published:** 2020-07-17

**Authors:** Irene Solés-Tarrés, Núria Cabezas-Llobet, David Vaudry, Xavier Xifró

**Affiliations:** ^1^New Therapeutic Targets Group (TargetsLab), Department of Medical Science, Faculty of Medicine, Universitat de Girona, Girona, Spain; ^2^Laboratory of Neuronal and Neuroendocrine Communication and Differentiation, Neuropeptides, Neuronal Death and Cell Plasticity Team, Normandie University, UNIROUEN, Inserm, Rouen, France

**Keywords:** Alzheimer’s disease, Parkinson’s disease, Huntington’s disease, cognition, synaptic plasticity, PAC1, VPAC1, VPAC2

## Abstract

Cognitive impairment is one of the major symptoms in most neurodegenerative disorders such as Alzheimer’s (AD), Parkinson (PD), and Huntington diseases (HD), affecting millions of people worldwide. Unfortunately, there is no treatment to cure or prevent the progression of those diseases. Cognitive impairment has been related to neuronal cell death and/or synaptic plasticity alteration in important brain regions, such as the cerebral cortex, substantia nigra, striatum, and hippocampus. Therefore, compounds that can act to protect the neuronal loss and/or to reestablish the synaptic activity are needed to prevent cognitive decline in neurodegenerative diseases. Pituitary adenylate cyclase-activating polypeptide (PACAP) and vasoactive intestinal peptide (VIP) are two highly related multifunctional neuropeptides widely distributed in the central nervous system (CNS). PACAP and VIP exert their action through two common receptors, VPAC1 and VPAC2, while PACAP has an additional specific receptor, PAC1. In this review article, we first presented evidence showing the therapeutic potential of PACAP and VIP to fight the cognitive decline observed in models of AD, PD, and HD. We also reviewed the main transduction pathways activated by PACAP and VIP receptors to reduce cognitive dysfunction. Furthermore, we identified the therapeutic targets of PACAP and VIP, and finally, we evaluated different novel synthetic PACAP and VIP analogs as promising pharmacological tools.

## Introduction

Cognition is the result of the formation of functional neuronal circuits in many cerebral areas governed by a dynamic phenomenon named synaptic plasticity. People suffering from neurodegenerative diseases commonly present cognitive impairment, such as dementia, deficits in learning and attention, or incomplete executive function among others, affecting the daily life of patients and their families. This impairment is mediated by the extracellular or intracellular accumulation of protein aggregates that disrupt synaptic plasticity leading to neuronal dysfunction and/or neuronal death. Several mechanisms have been involved in neuronal plasticity disturbance and neuronal cell death, such as inflammation, excitotoxicity, oxidative stress, and neurotrophic deprivation. These mechanisms affect genetic expression through the activation/inhibition of some pathways and transcription factors. The result of altered genetic expression is the disruption of structural and functional synaptic plasticity and/or the viability of neurons. Thus, molecules able to promote synaptic plasticity or neuronal viability by the interaction and inhibition of these mechanisms in brain areas related to learning and memory could be interesting therapeutic compounds to stop the cognitive decline in neurodegenerative diseases.

Pituitary adenylate cyclase-activating polypeptide (PACAP) and vasoactive intestinal polypeptide (VIP) are well-known neuropeptides that exert a potent neuroprotective effect through the activation of different signaling pathways and transcriptional-genetic activity (Dejda et al., 2005; Vaudry et al., 2009). Some data in animal models show that the lack of PACAP and VIP is associated with a cognitive decline. Indeed, PACAP deficient mice display impaired recognition memory (Shibasaki et al., 2015) and VIP deficient mice exhibit cognitive deficits (Chaudhury et al., 2008). Recently, it has been shown that VIP positive interneurons in the CA1 hippocampal area are necessary for spatial learning (Turi et al., 2019). Interestingly, in rat hippocampal slices, it has been observed that PACAP and VIP exert a direct effect in synaptic transmission (Ciranna and Cavallaro, 2003). This context and the capacity of PACAP and VIP to cross the blood-brain barrier suggest that these neuropeptides and their receptors-mediated signaling could have promising therapeutic activity in neurodegenerative diseases. In this review, we summarize the pieces of evidence showing that PACAP and VIP administration help to preserve cognitive function in different preclinical models of Alzheimer’s disease (AD), Parkinson’s disease (PD) and Huntington’s disease (HD). We also identify the main molecular mechanisms by which PACAP and VIP promote neuronal survival and synaptic plasticity in neurons to preserve cognitive performances. Finally, we review novel VIP and PACAP synthetic derivatives which could represent promising therapeutic tools for the treatment of neurodegenerative diseases.

## PACAP and VIP Promote Cognitive Function in Neurodegenerative Disease Models

### Alzheimer’s Disease

Alzheimer’s disease is the most common form of dementia characterized by the irreversible deterioration of cognitive function (Alzheimer’s Association, 2015) associated with the accumulation of amyloid-β (Aβ) plaques and neurofibrillary tangles in the cognitive brain areas (Hou et al., 2019).

The potential of PACAP as a therapeutic agent in AD neuropathology has been studied for many years in different experimental models (Table 1). *In vitro* studies have reported that PACAP protects against Aβ-mediated toxicity (Onoue et al., 2002; Han et al., 2014b). This neuroprotective effect of PACAP is associated with an improvement of the cognitive function in AD mice models. A long-term daily intranasal PACAP administration ameliorates the performance in the novel object recognition test in the AD transgenic mice model overexpressing the amyloid precursor protein (APP; Rat et al., 2011). In these animals, the rescue of memory deficits was linked with an increase of brain-derived neurotrophic factor (BDNF) and the enhancement of the non-amyloidogenic pathway of APP (Rat et al., 2011). Also, PACAP reduced the inflammatory response (Rat et al., 2011). Thus, PACAP is suggested to prevent cognitive decline in AD exerting neurotrophic, neuroprotective, and anti-inflammatory effects. Interestingly, the capacity of PACAP to counteract cognitive decline was also proved in SAMP8 mice, another AD model expressing the Aβ (Nonaka et al., 2012). In this study, PACAP alone or together with cyclodextrins was given by intranasal administration. PACAP was found in all brain regions, but some regions such as the occipital cortex and striatum incorporated much more PACAP than others. This pattern of distribution of the labeled PACAP is different from the one found when the peptide is given by intravenous injection, in which the highest uptakes are found in the hippocampus and hypothalamus (Nonaka et al., 2002). The addition of cyclodextrins can increase or decrease the uptake of PACAP in different brain regions depending on the cyclodextrin used. Such treatment could thus be used to promote neuropeptide uptake toward specific brain regions and was shown to improve the learning memory of SAMP8 mice in the T-maze task (Dogrukol-Ak et al., 2009). Because of these promising results, it was proposed that the downregulation of PACAP may be involved in AD neuropathology. Indeed, a comparative analysis of gene expression showed decreased protein levels of PACAP and BDNF in three different models of AD with Aβ deposition (Wu et al., 2006). Years later, PACAP expression was found decreased and inversely correlated with Aβ and Tau protein levels in a triple transgenic mouse model of AD (3xTG, Psen1/APPSwe/TauP301L; Han et al., 2014b). According to these findings, studies with AD patients demonstrated that PACAP levels were reduced in cortical areas such as the entorhinal cortex, the middle temporal gyrus, the superior frontal gyrus, and the primary visual cortex (Han et al., 2014a). Interestingly, PACAP deficits in humans were associated with clinical severity of AD (Han et al., 2015) and inversely correlated with Aβ plaques and neurofibrillary tangles (Han et al., 2014b). Altogether, these results point out that reduced levels of PACAP may contribute to the pathological process of AD and could have an important implication in the cognitive decline.

**Table 1:** Beneficial effects of pituitary adenylate cyclase-activating polypeptide (PACAP) and vasoactive intestinal peptide (VIP) in cellular and animal models of different neurodegenerative diseases.

		PACAP	VIP
Alzheimer’s disease	Animal model	Improves performance in the novel object recognition test in APP[V717I]-transgenic mice (Rat et al., 2011).	Decreases Aβ accumulation and atrophy in the hippocampus and cortex in 5XFAD mice (Korkmaz et al., 2019).
		Improves learning in T-maze task in SAMP8 mice (Dogrukol-Ak et al., 2009).	Reduces inflammation and attenuates amyloidosis in the hippocampus in PS1/APP transgenic mice (Song et al., 2012).
			Ameliorates the cognitive performance in the water maze test in ApoE mice (superactive VIP derivate; Gozes et al., 1997).
	Cellular model	Protects against Aβ-mediated toxicity in PC12 cells (Onoue et al., 2002) and primary neuronal cell culture (Han et al., 2014b).	Protects against Aβ-induced cell death in PC12 cells (Onoue et al., 2002) and cerebral cortex cell cultures (Gozes et al., 1996).
Parkinson’s disease	Animal model	Improves learning and memory in three different paradigms of water-maze task in MPTP-injected mice (Deguil et al., 2010).	Prevents from oxidative stress and apoptosis in 6-OHDA-lesioned rats (Tunçel et al., 2012).
		Prevents from motor and behaviour deficits in 6-OHA lesioned rats (Reglodi et al., 2004a,b).	Enhances the spine density and prevents from dopaminergic cell loss in 6-OHDA lesioned rats (Korkmaz et al., 2012).
		Improves locomotor function and behaviour alterations in rotenone-induced snails (Maasz et al., 2017).	Supresses microglial activation in MPTP- induced mice (Delgado and Ganea, 2003) and astrogliosis in 6-OHDA lesioned rats (Yelkenli et al., 2016).
			Reverses the rotational deficits in a 6-OHDA lesioned rats (Tunçel et al., 2005).
	Cellular model	Protects against 6-OHDA (Takei et al., 1998), MPTP (Chung et al., 2005), rotenone (Wang et al., 2005), salsolino (Brown et al., 2013) and paraquat (Hajji et al., 2019).	Protects against dopamine and 6-OHDA toxicity in PC12 and neuroblastoma cells (Offen et al., 2000).
Huntington’s disease	Animal model	Reduces the loss of striatal neurons and attenuates behavioural disturbances in striatum-lesioned mice by quinolonic acid injection (Tamás et al., 2006).
		Improves the performance in the novel object recognition test and the T-maze spontaneous alternation task in R6/1 transgenic mice (Cabezas-Llobet et al., 2018).	
	Cellular model	Induces neuritic branching in R6/1 hippocampal primary culture (Cabezas-Llobet et al., 2018).	

Different studies using animals and cell culture models of AD have revealed that VIP also acts as a neuroprotective agent against the pathogenesis of this disorder (Table 1). A recent study using 5XFAD mice, an AD model with massive cerebral Aβ deposits, shows that VIP intraperitoneal chronic administration significantly decreases Aβ accumulation and reduces the atrophy in brain regions involved in cognition, such as hippocampus and cortex (Korkmaz et al., 2019). Importantly, VIP protection against Aβ-induced cell death was also observed in experiments using PC12 cells and cortical cell cultures (Gozes et al., 1996; Onoue et al., 2002). However, the survival effect of VIP against Aβ-induced cell death seems to be less effective than PACAP protective activity. This result could be due to the growing evidence that the neuroprotective effect of VIP is mainly through its action on microglial cells. In microglia/neuron co-cultures, some studies showed that the presence of VIP protects from Aβ-induced neurodegeneration, inhibiting the secretion of pro-inflammatory interleukins and neurotoxic agents from microglia and inducing Aβ phagocytosis (Delgado et al., 2008; Song et al., 2012). Interestingly, both immunosuppressive and anti-amyloidosis effects of VIP have been confirmed *in vivo*. Constitutive overexpression of VIP in the hippocampus reduces inflammation and attenuates amyloidosis in transgenic PS1/APP mice, which present increased β-amyloid production associated with behavioral abnormalities (Song et al., 2012). Inflammatory reactions and microglia activation are well known to contribute to neuronal degeneration and to be associated with dementia in AD. Therefore, neuroprotective and immunomodulatory capacities of VIP make this neuropeptide an attractive candidate for the treatment of cognitive deficits in AD. However, the implication of VIP expression in AD pathology is still unclear. A study using human samples did not show any differences in VIP expression in different cognitive-related regions such as the hippocampus, amygdala, thalamus, and striatum of patients with AD (Ferrier et al., 1983). In contrast, a significant reduction of VIP immunoreactivity was found in the cerebral cortex of AD patients (Arai et al., 1984). In AD animal models, the role of VIP in the neuropathology has been poorly studied. Only in the AD mice model deficient in apolipoprotein E (ApoE), a reduction in VIP transcription was reported (Gozes et al., 1997). Interestingly, chronic intranasal administration of a superactive VIP agonist ameliorates the learning and memory deficits of these animals in water maze test (Gozes et al., 1997), suggesting that the peptide could at least be of therapeutical interest for the treatment of the disease.

### Parkinson’s Disease

Parkinson’s disease is the second most common neurodegenerative disorder after AD (Kalia and Lang, 2015). PD neuropathology is characterized by the degeneration of dopaminergic neurons in the substantia nigra pars compacta (SNc) and the subsequent loss of their projections to the striatum (Bezard et al., 2003). Although PD has long been characterized by motor disturbances, cognitive dysfunction is common in patients and can range from mild impairment to dementia (Caviness et al., 2007). Nowadays, the relationship between the neurodegenerative process and cognitive decline in PD remains unclear. While some authors point out a frontal lobe dysfunction because of the affection in the mesocortical dopaminergic system, others suggest a deficit in the cortico-basal ganglia circuit due to the nigrostriatal dopaminergic degeneration (Sawamoto et al., 2007). Besides, some clinical studies indicate that hippocampus atrophy could also contribute to memory impairment in PD (Brück et al., 2004).

The neuroprotective action of PACAP in PD has been well-established in both *in vitro* and *in vivo* models (Table 1, Reglodi et al., 2017, 2018). PACAP has proved to be neuroprotective in different cell cultures and explant models of PD, using 6-hydroxydopamine (OHDA), 1-Methyl-4-phenyl-1,2,3,6-tetrahydropyridine (MPTP), rotenone, salsolinol and paraquat neurotoxic agents (Takei et al., 1998; Chung et al., 2005; Wang et al., 2005; Brown et al., 2013; Lamine-Ajili et al., 2016; Hajji et al., 2019). These *in vitro* results are in line with findings observed in animal models, which have demonstrated the therapeutic action of PACAP in motor and behavioral disturbances of PD (Reglodi et al., 2004a,b; Maasz et al., 2017). The capacity of PACAP to prevent the cognitive impairment in PD was showed using three specific cognitive processes in an MPTP mouse model: habit learning, working memory, and spatial reference in learning and memory, which depend on the integrity of the striatum, frontal cortex, and hippocampus, respectively. PACAP could improve learning and memory functions in those three different paradigms (Deguil et al., 2010). Hence, the authors suggest that PACAP could not only restore the dopaminergic neurotransmission but also the hippocampal cholinergic transmission, as other authors had seen before (Masuo et al., 1993; Wang et al., 2008; Maasz et al., 2017). The fact that PACAP was able to protect the three neuronal cell types makes it a promising therapeutic agent to fight against cognitive decline in PD. Also, recent results obtained in different animal models of PD showed the dysregulation of PACAP and its receptors in brain areas related to cognition. A specific reduction of PAC1 receptor was reported in the basal ganglia of MPTP-induced parkinsonian macaques (Feher et al., 2018). Likewise, PACAP-knockout mice were shown to be more vulnerable to paraquat (a pesticide that increases the risk of PD) than wild-type (Watson et al., 2013). However, there are no studies relating to a decrease of PACAP or PAC1 receptor level with impairment of cognitive functions in PD. Therefore, further research is needed to establish if endogenous PACAP and its receptors are involved in the progression of PD, and specifically in cognitive symptoms.

In different PD neuronal and animal models, VIP has proved to protect either directly or indirectly (Table 1, Korkmaz and Tunçel, 2019). VIP was found to protect directly against dopamine and 6-OHDA toxicity in PC12 and neuroblastoma cells (Offen et al., 2000). VIP also exerted a direct effect preventing neurons from oxidative stress and apoptosis in a 6-OHDA murine model (Tunçel et al., 2012). Moreover, VIP was found to enhance the spine density and act as a neurotrophic factor in the striatum of parkinsonian rats (Korkmaz et al., 2012; Yelkenli et al., 2016). VIP could also indirectly exert protection through the suppression of both microglial activation and astrogliosis in PD (Delgado and Ganea, 2003; Yelkenli et al., 2016). Besides this, some authors support that VIP neuroprotective effect is probably mediated by masts cells, suggesting a VIP immunomodulatory action (Tunçel et al., 2005). Also, VIP was found to reverse the rotational deficits in a 6-OHDA rat model of PD (Tunçel et al., 2005), indicating a potential therapeutic activity. Unfortunately, no studies have been conducted regarding the specific effect on animals’ cognitive function. Moreover, in contrast to PACAP, there is no evidence that VIP participates in PD neuropathology. VIP levels were found unaltered in the brains of both demented and non-demented parkinsonian patients (Jégou et al., 1988).

### Huntington’s Disease

Huntington’s disease is a hereditary autosomal neurodegenerative disorder characterized by cognitive, psychiatric, and motor dysfunction. The genetic cause of HD is an abnormal expansion of CAG in the gene encoding for the protein huntingtin (htt; MacDonald et al., 1993). The resulting mutant htt (mhtt) protein causes a severe degeneration of striatal neurons that leads to motor disabilities. Also, mhtt promotes synaptic dysfunction in some cortical and hippocampal neuronal populations, which has been associated with the cognitive decline in HD (Giralt et al., 2012).

There are only two studies that explore the therapeutic potential of PACAP in the treatment of HD (Table 1). However, the results are promising. First, the effect of PACAP was studied in an excitotoxic model of HD. In HD-induced rats, PACAP treatment reduces the loss of striatal neurons and attenuates behavioral disturbances (Tamás et al., 2006). Years later, a second study found that PACAP can counteract the hippocampal-dependent cognitive decline in a transgenic HD mouse model by the analysis of novel object recognition test and the T-maze spontaneous alternation task (Cabezas-Llobet et al., 2018). This cognitive improvement is associated with an increased expression of proteins related to synaptic plasticity, such as BDNF, and the recovery of synaptic particles in the hippocampus. Moreover, in HD hippocampal primary cultures, PACAP treatment increases the number and length of neurites (Cabezas-Llobet et al., 2018). Thus, PACAP is suggested to improve the cognitive function by enhancing synaptic plasticity in the hippocampus. Interestingly, PACAP treatment also restores the PAC1 receptor protein level, which is altered in the hippocampus of HD mice from the onset of cognitive decline. Therefore, the beneficial effects of PACAP are suggested to be through the stimulation of the PAC1 receptor (Cabezas-Llobet et al., 2018).

Very few authors have studied the possible role of VIP in HD pathology. The first study regarding VIP and HD, showed no changes in VIP protein levels in the frontal cortex and basal ganglia in human HD post-mortem samples (Emson et al., 1979). However, some other studies indicate that VIP expression is altered in brain areas related to non-motor symptoms in HD. A post-mortem analysis of HD patients revealed a reduction of VIP immunoreactivity in the central nucleus of the amygdala (Zech et al., 1986). Recently, a decreased expression of VIP receptors was also found in the hippocampus of a transgenic HD mouse model (Cabezas-Llobet et al., 2018). Therefore, the impaired VIPergic signaling found in these regions could be involved in neuropathology which leads to cognitive and neuropsychiatric symptoms in HD (Fahrenkrug et al., 2007). Unfortunately, there are no other references regarding the relationship between VIP and non-motor disturbances in HD, and no information concerning the VIP therapeutic potential on the symptomatology.

### Amyotrophic Lateral Sclerosis and Multiple Sclerosis

Amyotrophic lateral sclerosis (ALS) and multiple sclerosis (MS) are two diseases that affect both the brain and spinal cord nervous tissue. ALS is marked by progressive degeneration of upper and lower motor neurons while MS is characterized by demyelination due to an autoimmune response. Although ALS and MS have been traditionally viewed as diseases of the motor system, cognitive impairment also occurs in these pathologies (Chiaravalloti and DeLuca, 2008; Benbrika et al., 2019). It has been suggested that VIP and PACAP may contribute to ALS and MS non-motor symptomatology (Staines, 2008). In ALS patients, VIP levels were found decreased in cerebrospinal fluid (CSF) while PACAP and PAC1 receptor had an altered expression in the motor cortex (Werdelin et al., 1989; Bonaventura et al., 2018). Similarly, in MS patients VIP and PACAP CSF levels are significantly diminished (Andersen et al., 1984; Baranowska-Bik et al., 2013). In animals, it has been shown that a synthetic modified analog of VIP exerts an anti-inflammatory effect improving motor function and increasing life-span in a ALS rat model (Goursaud et al., 2015). In an MS-related mouse model, PACAP ameliorated both clinical and pathologic manifestations of experimental autoimmune encephalomyelitis (EAE), while treatment with VIP reduced incidence and severity of EAE by an anti-inflammatory action (Kato et al., 2004; Fernandez-Martin et al., 2006). Moreover, PACAP deficient mice exhibited exacerbated EAE, a phenotype associated with increased inflammatory response, suggesting a protective role of the endogenous source of this neuropeptide in the disease (Tan et al., 2009, 2013). Unfortunately, no studies are exploring the capacity of PACAP and VIP to fight the cognitive decline in ALS and MS.

## Mechanisms of Action: from Receptor (PAC1, VPAC1 and VPAC2) Activation to Molecular Mechanisms

### PACAP/VIP Receptors and Its Distribution in the Central Nervous System

The beneficial effects of PACAP and VIP on cognitive disturbances that occur in neurodegenerative diseases are due to the expression of their receptors in affected brain areas. PACAP has a high and specific affinity for the PAC1 receptor (PAC1R; Harmar et al., 2012). Additionally, PACAP and VIP share two receptors: VPAC1 receptor (VPAC1R) and VPAC2 receptor (VPAC2R), thanks to their high homology of structure (~68% amino acid identity).

**Figure 1:**
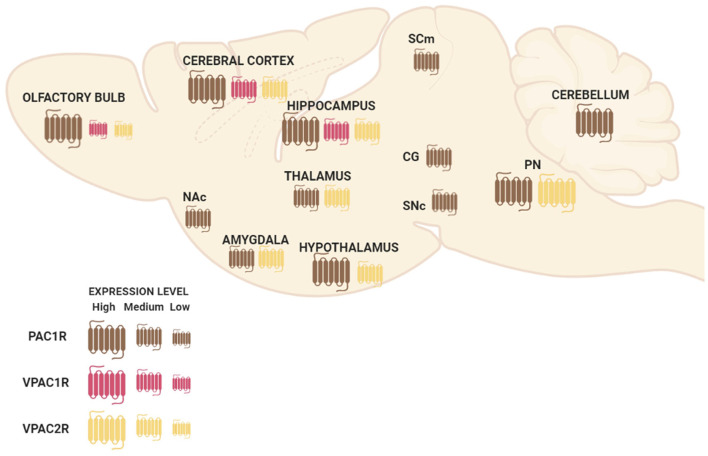
Schematic representation of pituitary adenylate cyclase-activating polypeptide (PACAP) and vasoactive intestinal peptide (VIP) receptors’ mRNA distribution in a sagittal view of the mouse brain. Abbreviations: Nac, nucleus accumbens; SCm, superior colliculus; CG, central gray; SNc, substantia nigra pars compacta; PN, pontine raphe nuclei.

PAC1R is much more expressed in the whole Central Nervous System (CNS) than VPAC1R and VPAC2R transcripts (Basille et al., 2000; Jolivel et al., 2009). The dentate gyrus of the hippocampus, the supraoptic nucleus of the hypothalamus, cerebral cortex, and olfactory bulb are regions where PAC1R is most highly expressed (Hashimoto et al., 1996; Nomura et al., 1996; Zhou et al., 2000). PAC1R mRNA high expression levels are also present in cingulate, entorhinal and piriform cortices; pyramidal and nonpyramidal cells of the hippocampal formation; amygdaloid nuclei; centromedial, mediodorsal, and ventromedial nuclei of the thalamus; hypothalamus; nucleus accumbens; superior colliculus, central gray and SNc; pontine raphe nuclei and cerebellum (Figure 1; Hashimoto et al., 1996; Shioda et al., 1996; Zhou et al., 2000). Concerning VPAC1R and VPAC2R, studies indicate a different but complementary distribution of their mRNA localization throughout the brain. VPAC1R is expressed in the hippocampus and the cerebral cortex, while VPAC2R highest expression is found in the thalamus, the hypothalamus, the hippocampus, the amygdala and the pontine nuclei (Figure 1; Vaudry et al., 2009). Both VPAC receptors have low expression in the olfactory bulb (Figure 1). In the cerebral cortex, VPAC1R is abundant in layers III and V, while on the other hand, VPAC2R is rather localized in layer VI (Usdin et al., 1994). Importantly, despite the distinctive mRNA distribution, protein expression of PAC1R, VPAC1R, and/or VPAC2R have been found in all cognition-related regions (Joo et al., 2004).

As all three receptors belong to the superfamily of G protein-coupled receptors, the beneficial effects of PACAP and VIP are associated to the activation of their related signaling second messengers: cAMP/adenylyl cyclase (AC)/protein kinase A (PKA), phospholipase (PLC)/protein kinase C (PKC), mitogen-activated protein kinase (MAPK) and phosphatidylinositol 3 kinase (PI3K)/Akt transduction pathways (Dickson and Finlayson, 2009; Vaudry et al., 2009). Expression and activity dysregulation of these proteins and related signaling mechanisms are involved in cognitive disturbances that occur in neurodegenerative diseases. Thus, the activation of these pathways and their related transcriptional machinery in brain areas related to learning and memory function has been proposed to favor the cognitive functions by promoting neuronal viability or synaptic plasticity (Giralt et al., 2012; Rai et al., 2019; Ureshino et al., 2019). Importantly, the actions of PACAP and VIP among these proteins can be direct or indirect, as receptors are not only present in neurons but are also expressed in glial cells (Ashur-Fabian et al., 1997; Grimaldi and Cavallaro, 1999).

### The anti-Apoptotic and Neurotrophic Functions of PAC1R

#### PAC1 Receptor in Anti-apoptotic Signaling

The antiapoptotic effects of PACAP in neurons are mainly mediated through PAC1R that activates pathways and genes downregulated in cognitive brain areas of neurodegenerative diseases. In immature cerebellar granule neurons, PACAP-PAC1R stimulation protects against many pro-apoptotic insults such as hydrogen peroxide, ethanol, and C2-ceramide (Vaudry et al., 2002b,c; Falluel-Morel et al., 2004; Aubert et al., 2006). This effect is associated with the AC/PKA pathway and phosphorylation of the extracellular signal-regulated kinase (ERK). Hence, PACAP action is blocked by PKA or MAPK inhibitors and mimicked by raising cAMP levels (Vaudry et al., 1998, 2000b; Falluel-Morel et al., 2004). Also, PAC1R activation promotes the transcription of the immediate-early gene c-fos, which induces B-cell lymphoma 2 (Bcl-2) expression. In the mitochondria, Bcl-2 blocks the cytochrome c release into the cytosol, preventing caspase-3 activation (Falluel-Morel et al., 2004; Aubert et al., 2006). Inhibition of mitochondrial apoptotic pathway by PACAP-PAC1R stimulation has been demonstrated *in vivo* using an AD mouse model overexpressing the APP. In these animals, PACAP intranasal administration increases both PACAP and PAC1R mRNA expression as well as the Bcl-2 protein levels (Rat et al., 2011). In addition to these findings, *in vitro* studies have demonstrated PACAP protective effect against Aβ-mediated toxicity *via* the inhibition of caspase-3 activity in PC12 cells (Onoue et al., 2002) or *via* the enhancement of the mitochondrial function in primary neuronal cell culture (Han et al., 2014b). Interestingly, in PC12 cells, it has been shown that PACAP-PAC1R activation also protects against rotenone-induced apoptosis, a PD cellular model, through a mitochondrial-independent pathway (Wang et al., 2005). Importantly, the molecular mechanisms involve the activation of MAPK by PKA leading to the inhibition of caspase-3 activity (Wang et al., 2005). PACAP also activates various genes through the PKA pathway (Vaudry et al., 2002a), some of which such as peroxiredoxin 2 (Botia et al., 2008), tissue plasminogen activator (tPA; Raoult et al., 2011), stathmin (Dejda et al., 2010) and Serpin b1a (Seaborn et al., 2014), being involved in its neuroprotective effect.

**Figure 2:**
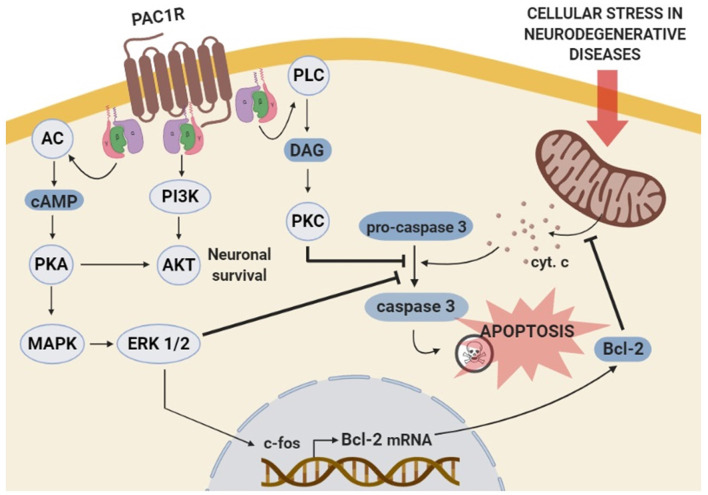
Schematic representation of the molecular mechanisms involved in PAC1R mediated anti-apoptotic effect. The stimulation of PAC1R activates protein kinase A (PKA) cascade signaling, leading to extracellular signal-regulated kinase (ERK) phosphorylation. Activated ERK induces the transcription of neuroprotective genes such as c-fos and Bcl-2, which finally inhibits the mitochondrial apoptotic pathway and blocks pro-caspase-3 activation. The inhibitory effect of the PACAP-PAC1R system on pro-caspase-3 cleavage can be induced via additional phosoholipase C (PLC)/protein kinase C (PKC) pathway. Moreover, PAC1R can also activate phosphatidylinositol 3 kinase (PI3K)/Akt system and promote neuronal survival to counteract the apoptotic process.

Besides AC/PKA and MAPK, some other pathways are suggested to be involved in the PAC1R-mediated anti-apoptotic action. For instance, PAC1R stimulation has demonstrated to prevent cerebellar granule neurons from caspase-3 activation *via* PKC transduction pathway (Vaudry et al., 2000a). In olfactory neurons, PAC1R also inhibits apoptosis *via* activation of the PLC (Han and Lucero, 2006). Moreover, PACAP protects against KCl-induced apoptosis of cerebellar granule neurons by the activation of the PI3K/Akt pathway (Bhave and Hoffman, 2003). In conclusion, PAC1R acts *via* different but sometimes complementary signaling pathways to block neuronal apoptosis mainly through the inhibition of caspase-3 activity (Seaborn et al., 2011; Figure 2). However, more data are needed to associate the activation of these pathways with the PACAP-mediated cognitive enhancement, especially since besides its direct neuroprotective effect, PACAP may act indirectly on neuronal survival *via* activation of PAC1R expressed by astrocytes. Indeed, PACAP stimulates endozepine release from cultured astrocytes *via* a PAC1R/PKA pathway (Masmoudi et al., 2003) and intracerebroventricular injection of the endozepine octadecaneuropeptide (ODN) prevents the degeneration of dopaminergic neurons in an *in vivo* model of PD (Bahdoudi et al., 2018). Interestingly ODN protects neurons from apoptosis through inhibition of the oxidative stress (Kaddour et al., 2013), which is often responsible for cell death in neurodegenerative diseases (Niedzielska et al., 2016).

#### PAC1 Receptor in Neurotrophic Signaling

PACAP/PAC1R stimulation has been found to enhance the expression of neurotrophins and related receptors in neuronal cells, promoting survival, synaptic plasticity, proliferation, and/or differentiation. Several *in vitro* and *in vivo* studies have shown that PAC1R stimulation can increase BDNF transcription and expression. BDNF is a key regulator of the maintenance of neuronal populations in the CNS and plays an important role in synaptic plasticity and synaptogenesis (Bramham and Messaoudi, 2005; Cohen-Cory et al., 2010; Bathina and Das, 2015). It has been demonstrated that BDNF protein level is positively correlated with the number of dendritic spines in the dentate granular neurons of mice (Stranahan, 2011). Importantly, BDNF expression is downregulated in many neurodegenerative diseases (Bathina and Das, 2015). It has also been shown a reduced expression of BDNF in PAC1R deficient-mice (Zink et al., 2004). Moreover, in AD and HD murine models and PD cellular models, beneficial effects of PACAP have been related to the increase of BDNF gene expression and protein levels (Rat et al., 2011; Brown et al., 2013, 2014; Cabezas-Llobet et al., 2018). Interestingly, some *in vitro* experiments have allowed the characterization of the molecular mechanisms involved. In rat cortical and hippocampal cultured neurons PACAP-PAC1R stimulation induced BDNF transcription through the activation of AC/PKA signaling pathway (Yaka et al., 2003; Dong et al., 2010). AC/PKA together with MAPK is known to act through phosphorylation of cAMP response element-binding protein (CREB), which enhances BDNF signal transduction (Impey et al., 1998; Tao et al., 1998). Accordingly, PAC1R activation has been shown to enhance CREB phosphorylation and BDNF expression in *in vitro* and *in vivo* neurodegenerative models (Rat et al., 2011; Brown et al., 2013; Cabezas-Llobet et al., 2018). Therefore, BDNF enhanced expression has been proposed to be a key mechanism underlying neuroprotection and cognitive improvement induced by PACAP-PAC1R stimulation.

**Figure 3:**
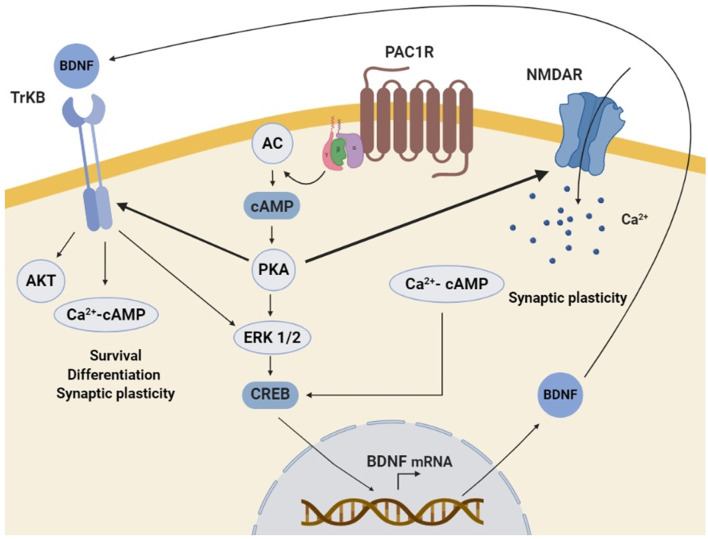
Schematic representation of the molecular mechanisms involved in PAC1R mediated neurotrophic effect. The stimulation of PAC1R by PACAP activates the protein kinase A (PKA) signaling pathway, which can potentiate N-methyl-D-aspartate (NMDA) receptors and/or induce downstream the phosphorylation of extracellular signal-regulated kinase (ERK). In both cases, CAMP response element-binding protein (CREB) is activated and enhances brain-derived neurotrophic factor (BDNF) transcription. Increased BDNF protein levels activate the TrkB receptor, which can also be potentiated by PKA. Finally, TrkB associated trophic signaling pathways promote neuronal survival, differentiation, and synaptic plasticity.

It has also been described that the capacity of PACAP-PAC1R to induce BDNF can be exerted through the glutamatergic N-methyl-D-aspartate (NMDA) receptor. NMDA receptors play an essential role in synaptic plasticity and synaptogenesis (Nicoll and Malenka, 1999). Dysregulation of NMDA receptors activity is associated with cognitive impairment in AD (Avila et al., 2017), PD (Vanle et al., 2018), and HD (Giralt et al., 2012) among others. Because their function is regulated by different cell modifications, the effect of PAC1R signaling on NMDA receptors activity has been studied. In cortical and hippocampal neurons PAC1R activation potentiates NMDA receptors, which induces BDNF expression (Pellegri et al., 1998; Yaka et al., 2003; Dong et al., 2010). It is described that NMDA receptors activation can promote the synthesis and release of BDNF (Marini et al., 1998). Therefore, PACAP/PAC1R has been proposed to regulate NMDA receptor activity promoting a trophic activity through the action of BDNF. Importantly, PAC1R related signaling can also activate directly the BDNF Tyrosine kinase B (TrkB) receptor, as observed in primary cultures of hippocampal neurons (Lee et al., 2002). Altogether, these results strongly suggest that PACAP-PAC1R enhances in a direct and/or indirect manner BDNF expression and trophic signaling responsible for neuroprotection, synaptic plasticity, and cognitive improvement (Figure 3).

PAC1R activation has also been shown to promote proliferation in neurogenic adult brain regions. This fact is interesting because, in the early stages of many neurodegenerative diseases, such as AD, HD, and PD, adult neurogenesis decreases, which is associated with non-motor symptoms such as memory impairment and cognitive decline (Winner et al., 2011). In adult mice, PAC1R stimulation was found to enhance the proliferation in lateral ventricle and dentate gyrus of the hippocampus, two neurogenic regions sensitive to neurodegeneration. The authors defined PKA as the principal signaling pathway involved (Mercer et al., 2004). In contrast, in immature cerebellar granule cells, PAC1R was suggested to inhibit proliferation *via* the AC/PKA pathway (Obara et al., 2007) responsible for the increase of Lot-1 expression (Contestabile et al., 2005; Fila et al., 2009). In cerebellar granule cells, PC12, and neuroblastoma cells, PAC1R activation promotes differentiation with neurite initiation and elongation (Deutsch et al., 1993; Hernandez et al., 1995; Gonzalez et al., 1997). Results of these studies point out an AC dependent but PKA independent signaling pathway in this action (Deutsch et al., 1993; Hernandez et al., 1995; Ravni et al., 2006, 2008). Moreover, a specific analysis of the transduction pathways involved in cell differentiation induced by PACAP concluded that PKC is not implicated (Vaudry et al., 1998). Downstream AC, PACAP would activate ras-proximate-1 (Rap1) and MAPK to induce early growth response protein 1 (Egr1) expression and in turn neuroblast differentiation (Ravni et al., 2008). The ability of PACAP to promote dendritic spine remodeling in cultured hippocampal neurons is mediated among others by the disintegrin and metalloproteinase 10 (ADAM10; Gardoni et al., 2012) and the microRNA-132 upregulation (Hayata-Takano et al., 2019). Taken together, these results indicate that, PAC1R activation coupled to AC and their downstream transduction pathways (Ravni et al., 2006) are determinant for the control of neuroblast proliferation and differentiation in brain regions affected by neurodegenerative diseases.

### Neuroprotective Actions Related to VPAC1R and VPAC2R

#### VPAC Receptors in Neurogenesis and Synaptic Function

VPAC receptors are considered dynamic regulators of postnatal and adult hippocampal neurogenesis by regulating survival, proliferation, and differentiation of precursor cells in the CNS. It has been shown that VPAC2R maintains stem cells population by supporting their survival and preventing neuronal or glial differentiation. In contrast, VPAC1R activation has been found to promote neurogenic granule cell fate (Zaben et al., 2009). Interestingly, both VPAC receptors were demonstrated to be determinant for the synaptic function in the hippocampus (Cunha-Reis et al., 2005). The activity of VPAC receptors has been associated with the PKA signaling pathway (Vaudry et al., 2000b) which leads to ERK activation, which finally promotes cell proliferation and/or differentiation (Langer, 2012). Moreover, the VPAC1R-mediated synaptic plasticity in the hippocampus is specifically mediated through the PKC pathway (Cunha-Reis et al., 2005).

#### VPAC Receptors in Glia Mediated Effects

Although some authors have found VIP direct effect in neurons, there is increasing evidence that most neuroprotective actions promoted by VPAC receptors in neurodegenerative diseases involve glial cells.

**Figure 4:**
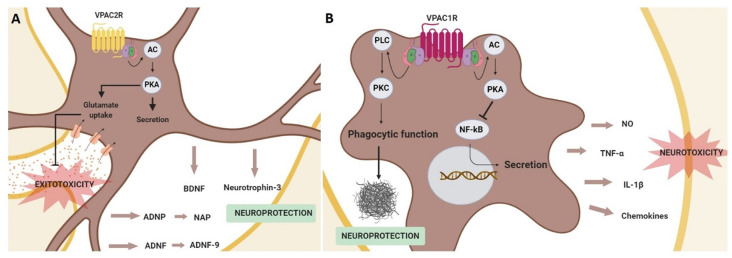
Schematic representation of neuroprotective effects mediated by the activation of the VPAC receptors in glial cells. In astrocytes **(A)** VPAC2R activation promotes the secretion of neurotrophic and neuroprotective factors, in addition to prevent neurons from excitotoxicity by enhancing the glutamate re-uptake in astrocytes. These actions are associated with adenylyl cyclase (AC)/protein kinase A (PKA) activation. In microglia **(B)** the stimulation of VPAC1R inhibits the secretion of pro-inflammatory agents through the activation of the PKA signaling pathway and protects against β-amyloid fibrils enhancing the microglia phagocytic function *via* protein kinase C (PKC) activation.

In astrocytes, it has been demonstrated that VPAC receptors activation induces the secretion of neurotrophic and neuroprotective factors (Figure 4). For instance, activity-dependent neuroprotective protein (ADNP) and activity-dependent neurotrophic factor (ADNF) are released after VPAC receptors stimulation from astrocytes (Brenneman and Gozes, 1996; Bassan et al., 1999). ADNP and ADNF regulate brain development and promote neuronal survival and plasticity (Brenneman and Gozes, 1996; Gozes and Spivak-Pohis, 2006). Importantly, these functions are mainly related to their most active fragments, NAP (NAPVSIPQ) and ADNF-9 (SALLRSIPA), respectively, which are demonstrated to be neuroprotective against multiple toxins *in vitro* (Gozes et al., 2003; Lagrèze et al., 2005; Smith-Swintosky et al., 2005). For instance, both ADNF9 and NAP protect against dopamine and 6-OHDA toxicity in PC12 and neuroblastoma cell models of PD (Offen et al., 2000). Interestingly, VIP-stimulated astrocytes can also secret neurotrophin-3 (Brenneman et al., 1997, 2003; Dejda et al., 2005). Specific involvement of VPAC1R and VPAC2R in astrocyte-mediated neuroprotection has been poorly studied, but some results suggest that VPAC2R plays the main key role. It has been found that VIP/VPAC2R system is neuroprotective by promoting the release of ADNP and BDNF in cultured astrocytes (Zusev and Gozes, 2004; Passemard et al., 2011). Moreover, the increased expression of glutamate transporters associated with reactive astrocytes could prevent cultured neurons against excitotoxicity (Nishimoto et al., 2011). Importantly, astrocytic-dependent neuroprotection has been related to the cAMP/PKA pathway (Nishimoto et al., 2011; Passemard et al., 2011; Figure 4).

It has also been described that VIP can regulate the release of proinflammatory cytokines from microglia. This action of VIP on microglial cells has been studied in neurodegenerative diseases, as excessive microglial activation contributes to the physiopathology of AD, PD, and HD, among others (Subhramanyam et al., 2019). In AD and PD models, it has been shown that VIP exerts a neuroprotective effect through the inhibition of tumor necrosis factor-α (TNFα), interleukin 1β (IL-1β), nitric oxide (NO) and different chemokines release (Delgado, 2002; Delgado and Ganea, 2003; Delgado et al., 2008; Song et al., 2012). Importantly, this VIP effect has been related specifically to VPAC1R activation coupled to the cAMP/PKA pathway and associated to the inhibition of NF-κB activation, a transcription factor of several genes encoding neurotoxic mediators (Delgado, 2002; Delgado and Ganea, 2003; Delgado et al., 2008). Interestingly, in a AD *in vivo* model, VPAC1R stimulation also enhances microglial phagocytosis of fibrillar beta-amyloid, but in this case through the activation of the PKC pathway (Song et al., 2012; Figure 4).

Even if the above information suggests a key role of VPAC receptors coupled to the cAMP/PKA pathway in the regulation of adult neurogenesis most of the actual investigations are restricted to their function during development of the SNC. Consequently, more studies are needed to clarify and understand the contribution of VPAC receptors in the context of cognitive dysfunction in neurodegenerative diseases. Currently, it is considered that in the context of neurodegenerative diseases, the VIP-VPACR system exerts its neuroprotective action mainly indirectly through astroglial and microglial cells. In astrocytes, VPAC2R has a key role in promoting the secretion of different neuroprotective and neurotrophic factors, besides preventing from glutamate excitotoxicity. On the other hand, VPAC1R in microglia acts as an inflammatory mediator and protects against β-amyloid fibrils.

## Designing PACAP and VIP Synthetic Derivates to Improve The Therapeutic Activity in Neurodegenerative Diseases

The therapeutic application of PACAP and VIP neuropeptides for the treatment of neurodegenerative diseases shows some limitations. Importantly, both neuropeptides are susceptible to be rapidly degraded. In humans, PACAP38 isoform has a plasma half-life of less than 5–10 min (Ramos-Álvarez et al., 2015) while VIP shows a plasmatic half-life of approximately 1 min (Domschke et al., 1978). Additionally, the widespread distribution of PACAP/VIP receptors in the CNS and the peripheral tissues can cause many side effects. Therefore, recent developed synthetic analogs showing increased plasmatic half-life and displaying receptor selectivity have been proposed as therapeutic agents.

PACAP is metabolized mainly by dipeptidyl peptidase IV (DPP IV; Zhu et al., 2003), which promotes the formation of dipeptides from the N-terminus (Mentlein, 1999). As the PACAP amino-terminal domain is essential for the activation of its receptors (Gourlet et al., 1996), the cleavage by DPP IV suppresses PACAP biological activity (Gourlet et al., 1996). Moreover, PACAP can also be cleaved by endopeptidases, that recognize two dibasic pairs (Arg14-Lys15 and Lys20-Lys21), and carboxypeptidases, which action depends on the C-terminal segment (Bourgault et al., 2009). Therefore, chemical strategies aimed to modify the putative DPP IV, endopeptidase, and carboxypeptidase sites of cleavage offer the possibility to obtain more stable PACAP-analogues. For instance, the addition of an acetyl or hexanoyl group at the N-terminus and the inversion of chirality of the Ser residue in position 2 are known to offer stability to PACAP by completely suppressing the action of DPP IV (Bourgault et al., 2008). Additionally, different amino acids have been determined as crucial for the specific binding of PACAP to its receptors, including His1, Asp3, and Phe6 (Lamine et al., 2016). As PACAP neuroprotective effects are mainly related to PAC1R, it has been proposed synthetic PAC1R agonists as interesting therapeutic molecules (Ramos-Álvarez et al., 2015). The Acetyl-[Ala15, Ala20] PACAP38 propylamine was designed to be a potent PAC1R agonist, in addition, to be more metabolically stable than native PACAP (Bourgault et al., 2008). Recently, the effects of this analog on memory and post-learning BDNF expression have been studied in Wistar rats. Surprisingly, the administration of acetyl-(Ala15, Ala20) PACAP38-propyl amide did not improve the cognitive function of rats, and accordingly, did not induce BDNF expression (Ladjimi et al., 2019). However, the authors showed that this analog could increase the activity of antioxidative enzymes in the neocortex of rats (Ladjimi et al., 2019). Unfortunately, the effect of acetyl-(Ala15, Ala20) PACAP38-propyl amide has not been tested in neurodegenerative models. The Ac-[Phe(pI)6, Nle17] PACAP (1–27) is another analog designed to specifically activate PAC1R/VPAC1R without affinity to VPAC2R. This is important because the activation of VPAC2R is associated with peripheral side effects such as vasodilation, an increase of heart rate, and water retention (Warren et al., 1992; Tsutsumi et al., 2002; Farnham et al., 2012). Interestingly, Ac-[Phe(pI)6, Nle17] PACAP (1–27) presents resistance against dipeptidyl peptidase IV activity, increasing its stability in human plasma (Lamine et al., 2016). This analog protects from MPP^+^-induced toxicity and appeared to be as efficient as PACAP (Lamine et al., 2016). Moreover, it was found to exert reduced cardiovascular side effects after treatment in an *in vivo* model of PD. However, the capacity of Ac-[Phe(pI)6, Nle17] PACAP (1–27) analog on cognitive deficits in neurodegenerative models has not been evaluated yet. Shorter fragments such as the amidated PACAP23 have shown their neuroprotective action *in vitro* (Lamine et al., 2019) but it remains to investigate if they keep their activity *in vivo*, in particular after intranasal injection. Indeed, the C terminal part of PACAP seems to be important for its transportation across the blood-brain barrier (Banks et al., 2006). Thereby, novel PACAP-TAT peptide with enhanced ability to cross biological barriers (Yu et al., 2012) and gH625-liposomes (Iachetta et al., 2019) may help to deliver PACAP derivatives to the brain.

Regarding VIP, its susceptibility to endopeptidases is also well known (Deng and Jin, 2017). To overcome this metabolic limitation, the addition of an N-terminal stearic acid (lipophilic) to different VIP short fragments was tested. The sequence stearyl–Lys–Lys–Tyr–Leu–NH2 (stearyl–KKYL–NH2) was found to have higher stability than VIP, providing the main neurotrophic and neuroprotective actions of the peptide (Gozes et al., 1999). Moreover, replacing the methionine in position 17 with a norleucine (Nle), resulted in a superactive VIP analog. Named stearyl-Nle17-VIP (SNV), it is 100-fold more potent than VIP in promoting neuronal survival, acting at femtomolar-picomolar concentrations in rat cerebral cortical cultures (Gozes et al., 1995; Ashur-Fabian et al., 1999). Interestingly, the beneficial effects of SNV in cognitive function have been tested *in vivo*. Using two rat models of developmental retardation, water maze experiments showed that SNV improves cognitive functions (Gozes et al., 1998). Similarly, chronic treatment with SNV was demonstrated to improve learning and memory functions in ethylcholine aziridium treated animals (Gozes et al., 1996) and in an apolipoprotein E (ApoE) deficient mice (Gozes et al., 1997), used as models of AD. For its part, the VPAC2R agonist D-p-Cl-Ac-Phe(pI)6, Leu17-VIP slows the pathogenesis of PD through modulation of the inflammatory response (Olson et al., 2015).

Altogether, several analogues more stable and selective than PACAP and VIP have been designed to increase the therapeutic activity and applicability. However, their role in cognitive dysfunction in neurodegenerative diseases is poorly known. Therefore, further research and some clinical trials are needed to ensure the therapeutic potential of these promising analogs.

## Author Contributions

IS-T, NC-L, and XX wrote the manuscript. IS-T, DV, and XX revised the literature. Supervision and conceptualization was performed by DV and XX. Editing was performed by XX.

## Conflict of Interest

The authors declare that the research was conducted in the absence of any commercial or financial relationships that could be construed as a potential conflict of interest.
